# Supplementary figures and images for: Temporal dynamics of SARS-CoV-2 shedding in feces and saliva: a longitudinal study in Norfolk, United Kingdom during the 2021–2022 COVID-19 waves
Source: Microbiol Spectr. 2025 Mar 25;13(5):e03195-24. doi: 10.1128/spectrum.03195-24 (PMC12053912; doi:10.1128/spectrum.03195-24)

# Supplementary Figure

#
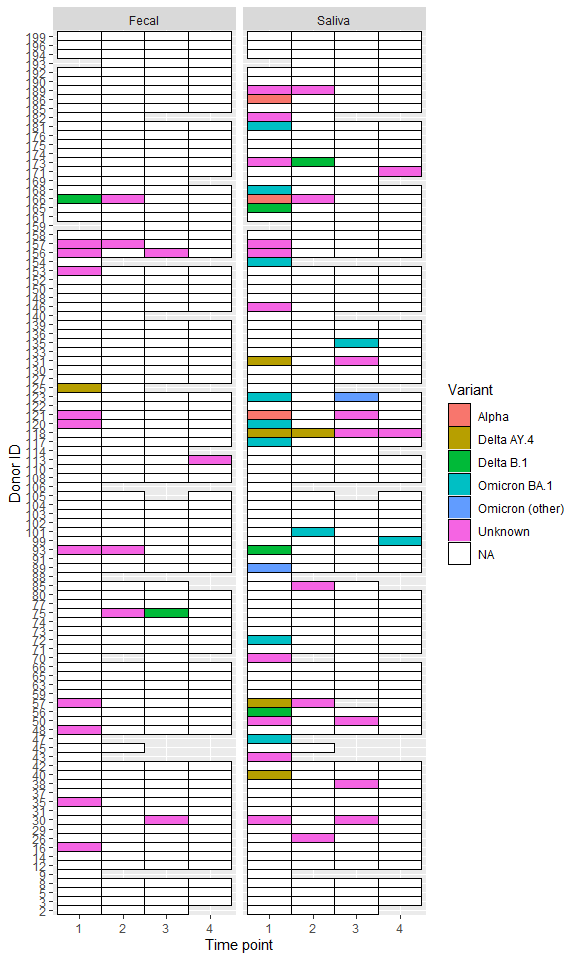


**Supplementary Figure 1:** Distribution of positive samples and variants.

Supplement: Fig. S1 — Distribution of positive samples and variants. [file spectrum.03195-24-s0001.docx]
